# Mobile Technology for Community Health in Ghana: Is Maternal Messaging and Provider Use of Technology Cost-Effective in Improving Maternal and Child Health Outcomes at Scale?

**DOI:** 10.2196/11268

**Published:** 2019-02-13

**Authors:** Michelle Willcox, Anitha Moorthy, Diwakar Mohan, Karen Romano, David Hutchful, Garrett Mehl, Alain Labrique, Amnesty LeFevre

**Affiliations:** 1 Johns Hopkins Bloomberg School of Public Health Baltimore, MD United States; 2 Grameen Foundation Ghana Accra Ghana; 3 World Health Organization Geneva Switzerland; 4 Health Intelligence Initiative Division of Epidemiology and Biostatistics, School of Public Health and Family Medicine University of Cape Town Cape Town South Africa

**Keywords:** child health, frontline health workers, Ghana, health information systems, maternal health, mHealth, mobile phone, newborn health

## Abstract

**Background:**

Mobile technologies are emerging as tools to enhance health service delivery systems and empower clients to improve maternal, newborn, and child health. Limited evidence exists on the value for money of mobile health (mHealth) programs in low- and middle-income countries.

**Objective:**

This study aims to forecast the incremental cost-effectiveness of the Mobile Technology for Community Health (MOTECH) initiative at scale across 170 districts in Ghana.

**Methods:**

MOTECH’s “Client Data Application” allows frontline health workers to digitize service delivery information and track the care of patients. MOTECH’s other main component, the “Mobile Midwife,” sends automated educational voice messages to mobile phones of pregnant and postpartum women. We measured program costs and consequences of scaling up MOTECH over a 10-year analytic time horizon. Economic costs were estimated from informant interviews and financial records. Health effects were modeled using the Lives Saved Tool with data from an independent evaluation of changes in key services coverage observed in Gomoa West District. Incremental cost-effectiveness ratios were presented overall and for each year of implementation. Uncertainty analyses assessed the robustness of results to changes in key parameters.

**Results:**

MOTECH was scaled in clusters over a 3-year period to reach 78.7% (170/216) of Ghana’s districts. Sustaining the program would cost US $17,618 on average annually per district. Over 10 years, MOTECH could potentially save an estimated 59,906 lives at a total cost of US $32 million. The incremental cost per disability-adjusted life year averted ranged from US $174 in the first year to US $6.54 in the tenth year of implementation and US $20.94 (95% CI US $20.34-$21.55) over 10 years. Uncertainty analyses suggested that the incremental cost-effectiveness ratio was most sensitive to changes in health effects, followed by personnel time. Probabilistic sensitivity analyses suggested that MOTECH had a 100% probability of being cost-effective above a willingness-to-pay threshold of US $50.

**Conclusions:**

This is the first study to estimate the value for money of the supply- and demand-side of an mHealth initiative. The adoption of MOTECH to improve MNCH service delivery and uptake represents good value for money in Ghana and should be considered for expansion. Integration with other mHealth solutions, including e-Tracker, may provide opportunities to continue or combine beneficial components of MOTECH to achieve a greater impact on health.

## Introduction

Globally, an estimated 273,500 maternal and 6.3 million child deaths occur each year; nearly half are in sub-Saharan Africa [1,2]. Ghana, home to 24.5 million people, is estimated to have 3,100 maternal and 56,000 child deaths annually [3]. While maternal and child mortality rates have both declined by 30% between 2000 and 2012, the rate of reduction annually over the last decade has not been enough for Ghana to achieve the Millennium Development Goals’ targets for maternal and child health [3].

Public health programs that aim to capitalize on mobile health (mHealth) technologies are emerging as potentially novel and effective strategies to address critical health system constraints [4-7]. Over the last decade, more than 600 documented mHealth strategies and comprehensive programs have been introduced globally, and innumerable small-scale pilots are in development or underway [8]. Ghana has seen a similar proliferation of mHealth programs alongside increasing rates of phone usage in the population: at least 22 different projects have been piloted since 2004 [5,9]. While evidence exists on the effectiveness of mHealth programs in improving health outcomes, fewer studies have investigated the cost-effectiveness of these new technology-supported solutions [7,10-14]. While there is broad consensus on the potential for mHealth solutions to improve the management of health systems, provider performance, and empower clients [15], several technical complexities, including network coverage, technology use, and data flow, when coupled with limited information on the effectiveness and cost-effectiveness, have impeded efforts to sustain and scale programs in areas with limited resources.

The Mobile Technology for Community Health (MOTECH) program aims to empower pregnant women and new mothers to improve knowledge and utilization of services through mobile phone-based voice messages on maternal, newborn, and child health (MNCH) and system-managed appointment reminders and alerts for important care visits. Simultaneously, MOTECH supports frontline health workers in collecting patient-level clinical information, improving data-reporting processes, and improving the timeliness and continuity of MNCH service delivery for clients in their catchment area [16,17]. The program was first initiated in 2009 in community-level health facilities in the Upper East Region of Ghana and later expanded to the Central, Greater Accra, and Volta Regions. To date, implementation has occurred in all public health centers and rural frontline health facilities in 7 districts: 24 health centers and 83 Community-based Health Planning and Services (CHPS) facilities.

To complement efforts to evaluate the effectiveness of MOTECH [18,19], we sought to determine the incremental cost-effectiveness of MOTECH as compared to existing health services in 170 districts over a 10-year analytic time horizon (2015-2024). Study findings aim to support evidence-based decision making on the value for money of MOTECH. This is the first study of its kind on the cost-effectiveness of an mHealth solution in Ghana and one of few globally on mHealth strategies for enhancing community-based MNCH services through the support of frontline health workers [20].

## Methods

### Study Setting

Ghana’s estimated population of 24.5 million is spread across 216 districts. Health services in the public sector are delivered through community CHPS facilities, health centers, and district and regional hospitals with varying rates of public sector service utilization. MNCH indicators have improved with the help of policies such as the Free Maternal Care Policy and National Health Insurance; however, maternal mortality (380 per 100,000 live births) and infant mortality (49 per 1000 live births) rates did not decrease sufficiently to reach 2015 targets, and socioeconomic and geographic differences continue to reflect inequalities in health service utilization and outcomes [1,3,21]. Utilization of at least 1 antenatal care (ANC) session is nearly universal, and 84% of women attend 4 or more ANC visits, nearly 30% of deliveries are not attended by a skilled birth attendant, and almost 20% do not receive postnatal care [22].

In an effort to catalyze improvements in the quality and continuity of care during pregnancy and postpartum, the MOTECH program launched in August 2010 in Kassena-Nankana West District in the Upper East Region of Ghana and in 2011 in Awutu Senya District in the Central Region. In 2012, with added funding from United States Agency for International Development and the Bill and Melinda Gates Foundation (BMGF), MOTECH was expanded into new districts: Dangme East in Greater Accra, South Tongu in the Volta region, and Gomoa West in Central region. Gomoa West was selected for an independent evaluation led by Healthcare Innovation Technology Lab in partnership with the University of Ghana School of Public Health. Because of the availability of data on health effects, data from Gomoa West were used in this analysis to forecast the costs and consequences of program expansion to 170 districts across Ghana.

Gomoa West is a semirural coastal district of the Central Region with a population of 135,139 [23]. Health services in Gomoa West are delivered through 17 CHPS facilities (1 per 7949 population), 4 health centers (1 per 33,785 population), and 1 district hospital [16]. Population and health systems characteristics for the remaining 169 districts were drawn from the 2010 Population and Housing Census, including district-division and population statistics, data on the number of health facilities, and human resources [23,24].

### Ethical Review

This study received ethical approval from the Ethical Review Committee of Ghana Health Service (GHS) as well as the Johns Hopkins School of Public Health Institutional Review Board. Requirement of informed consent was waived.

### Program Description

MOTECH, a partnership between Grameen Foundation and GHS, aims to improve service delivery and access to MNCH services through 2 components: Mobile Midwife and Client Data Application (CDA). Mobile Midwife is a demand-side intervention, which aims to improve client knowledge and awareness of key health information during pregnancy and postpartum, with the goal of stimulating best practices and encouraging timely and appropriate service utilization. Under Mobile Midwife, registered pregnant women and mothers of infants are provided with a MOTECH identification number and receive weekly local-language interactive voice response messages on pregnancy and infant care and appointment reminders for routine clinical visits on their mobile phones (Multimedia Appendix 1). To complement demand generation through Mobile Midwife, the CDA of MOTECH allows frontline health workers affiliated with CHPS facilities and health centers to use mobile phones to digitize clinical care information to better track and deliver care to women of reproductive age, including pregnant women and children <5 years of age. CDA consists of simplified digital registers consolidating information previously collected in over a dozen paper-based registers in health centers and CHPS facilities. Data elements recorded onto mobile phones are used to generate monthly reports as well as care reminders and alerts sent to both the clients and nurses along with a weekly list of care defaulters in need of follow-up in their catchment area [16]. The input of digitized health data activates the automated system to cross-reference national care protocols, identify appropriate reminders or alerts to clients and nurses, and send voice messages. The MOTECH program also facilitates data reporting by summarizing facility statistics for a report to the district, although the reporting system is not directly interoperable with the government health information system.

As part of this analysis, we forecasted the costs and consequences of scaling up MOTECH activities to 170 districts across Ghana, mirroring the implementation activities conducted in Gomoa West. Program activities were provided in 3 phases: development (6 months), start-up (6 months), and ongoing support to implementation (Table 1). Development encompasses all central activities, such as national stakeholder meetings, which are necessary to prepare for the initiation of the program. Start-up includes all central- and local-level activities required to initiate the program, including district profiling, content localization, orientations, and training. Implementation refers to ongoing activities required to sustain the program and includes salary support for personnel at all levels for ongoing monitoring and evaluation as well as recurrent telecommunications and data usage costs, which assume no fluctuation for agreements with mobile network operators from what was established in the MOTECH program in Gomoa West. Development and start-up phase activities occur at the program’s inception and are considered capital investments that are annualized across the life of the program. Some activities were modified for national scale from the original Grameen Foundation MOTECH project in Gomoa West: instead of mass registration campaigns for enrollment, the national program would assume point-of-care enrollment at health facilities. Recurring costs such as personnel, utilities, and vehicle maintenance are included in all phases.

To initiate MOTECH activities at scale, a 3-year national rollout plan was designed to begin in 3 regions, with 5 districts in each region conducting start-up activities simultaneously supported by key central and regional support staff for training, program administration, and technical support (Table 2). Additional districts could be brought on board within those regions once the first districts completed all development activities and had moved on to start-up or implementation phases of the program. GHS successfully upgraded from District Health Information System (DHIS) version 1 to the current DHIS 2 in only 6 months, and although this is promising for the capacity and experience with digital health solutions, the roll out of MOTECH activities at scale would require significant oversight capacity for additional health worker training, equipment replacement, and data monitoring [25].

**Table 1:** Program costs by phase and activity for the gradual rollout of Mobile Technology for Community Health (MOTECH) to 170 districts from 2015-2024.

Program activities		National program activity description and input	Total cost (US $)
**Development^a^**			
	Program design	National leadership meetings among central program leadership, regional, and district health management teams	36,800
	Telecommunications	Voice message program national negotiations and ongoing partnership with telecommunications companies, infrastructure establishment to manage call system	23,480
	Technology	Platform support and server hosting	16,907
	Personnel	Central, regional, and district staff time allocated to develop the program for each district	596,955
	Subtotal development		698,647
	Subtotal development costs per district per year		4,110
**Start-up^b^**			
	District profiling	Profile data on health system facilities and telecommunication infrastructure compiled by district health management teams	150,919
	Content localization	Voice health messaging content standardized, translated, recorded, and tested for 9 recognized national languages	30,690
	Equipment	Phone purchases for facilities to use for MOTECH mHealth application platform, additional staff equipment	2,471,799
	Customer support	Customer service referral system for technical or programmatic issues	249,746
	Training	Orientation for leadership, nurse training on simplified registers and data entry application, and subdistrict program orientation	2,406,578
	Community mobilization	District launch events, durbars, or other marketing	627,638
	Partnership building	Regional steering committee meeting for district program planning and technical steering committees in the district meet monthly for 4 months to direct start-up	10,449
	Vehicle maintenance	Cost to maintain and use existing vehicles without new capital cost purchase	172,082
	Office maintenance	Central office space	86,277
	Telecommunications	Airtime for voice messages, nurse data upload cost	77,166
	Technology	Platform support, server hosting, and system scaling by region for increased call capacity	26,318
	Personnel & benefits	Central, regional, and district staff time allocated to initiating the program for each district	1,193,847
	Subtotal start-up		7,503,508
	Subtotal start-up costs per district per year		44,138
**Implementation^c^**			
	Technical groups	District health management teams will include program tasks in current workflow, no added cost	17,810
	Monitoring and evaluation	Routine data entry application use monitoring and data validation	1,700,567
	Continued training	Refresher training and training of new hires	2,140,605
	Equipment & materials	Phone replacement and simplified registers annual set per facility	4,928,480
	Vehicle Maintenance	No vehicle cost in national program	1,078,131
	Field office maintenance	District office space	209,521
	Office maintenance	Central office space	257,036
	Telecommunications	Airtime for voice messages and nurse data upload cost	2,343,275
	Technology maintenance	Data platform and information technology technical assistance	241,582
	Personnel & benefits	Central, regional, and district staff time allocated to maintaining the program	11,218,454
	Subtotal implementation		24,135,461
	Subtotal implementation costs per district per year		17,918
	Total annualized cost per district per year		66,166

^a^6 months in duration per district.

^b^6 months in duration per district.

^c^9 years of implementation annual costs for 45 districts in which MOTECH program activities were initiated in 2015, 8 years for 67 districts in which MOTECH program activities were initiated in 2016, 7 years for 58 final districts in which MOTECH program activities were initiated in 2017.

**Table 2:** Rollout plan for the Mobile Technology for Community Health program: Reaching 170 districts over 3 years.

Ghana regions	Number of districts						
	Year 1 January-June	Year 1 July-December	Year 2 January-June	Year 2 July-December	Year 3 January-June	Year 3 July-December	Total
Western	5	5	7	—^a^	—	—	17
Central	5	5	7	—	—	—	17
Greater Accra	5	5	—	—	—	—	10
Volta	—	5	5	8	—	—	18
Eastern	—	5	5	5	6	—	21
Ashanti	—	5	5	5	5	7	27
Brong-Ahafo	—	—	5	5	5	7	22
Northern	—	—	5	5	5	5	20
Upper East	—	—	—	—	5	4	9
Upper West	—	—	—	—	5	4	9
New districts starting up	15	30	39	28	31	27	—
All districts with program costs	15	45	84	112	143	170	170

^a^No start-up costs were being incurred in this district.

### Costs

Economic costs were estimated from a program perspective for a 10-year analytic time horizon (2015-2024). Program costs were defined as the costs required to develop, start up, and support ongoing implementation. Costs associated with MOTECH activities from 2012-2014 in the district of Gomoa West were used to generate estimates of the resources required to scale up and sustain implementation into 170 districts. Drawing from Grameen Foundation project financial records in Accra, which detailed capital and recurrent expenditures over time for MOTECH in Gomoa West, as well as informant interviews with program staff, we used an ingredients approach to forecast estimates of costs by activity and level of the health system (central, regional, or district) over time for each district. District population estimates were drawn from the 2010 census in Ghana and the model adapted to incorporate differences across districts in numbers of CHPS facilities and health centers [23]. Once collected, costs were converted into US $ , using month-appropriate market exchange rates, and then adjusted into 2014 US $ using the 2014 Consumer Price Index for Ghana [26]. Capital costs were annualized over the lifetime of the project or life span of the item as appropriate and discounted at 3% [27]. Development and start-up phase costs were viewed as one-time activities and similarly annualized over the lifetime of the project. Variable costs were anticipated to change with the number of facilities or nurses (mobile phones purchased, refresher training, etc) and adjusted based on population rates in Gomoa West.

### Health Effects

Health effects were modeled based on the average intervention effects observed as part of the independent evaluation of MOTECH carried out in Gomoa West and 1 comparison district. Secondary analyses of exit interview data conducted by our team sought to generate an estimate of the average treatment effect of the MOTECH program based on propensity score values. Findings from this analysis suggested a significant increase in the coverage of skilled birth attendants (11%), facility delivery (10%), and measles immunization (6%) following program implementation. No significant changes were observed in pregnant women reporting 4 or more ANC visits, exclusive breastfeeding, the use of modern methods postpartum family planning, and other indicators. Indicators with significant increases in health effects were inputted in to the Lives Saved Tool (LiST) in Spectrum version 4.7. Increases in coverage were assumed to increase linearly but not anticipated to increase at the same rate over time. When intervention coverage surpassed 75%, the rate of coverage increase was adjusted by half; above 90% overall intervention coverage, the rate of increase is again halved; and for any intervention, cannot increase beyond 99%. These breakpoints help to adjust expectations of increases in coverage with the model’s time horizon. The disability-adjusted life years (DALYs) averted were calculated with a 3% discount rate, no age weighting, and life expectancy of 65.5 years as reported in the 2010 Census [23,28]. In the absence of data on disabilities, DALYs are based on years of life lost only.

### Analyses

Analyses of incremental costs and effects were conducted in Microsoft Excel. To test for uncertainty, one-way and probabilistic sensitivity analyses were conducted. The latter was carried out in Microsoft Excel using Monte Carlo simulation with 1000 iterations per analysis. The resulting mean point estimate was obtained by dividing mean costs by mean effects and presented along with the 95% CI. A cost-effectiveness plane and cost-effectiveness acceptability curve (CEAC) were used to calculate the probability that the intervention would be cost-effective for each of a number of standard thresholds of cost-effectiveness. Cost-effectiveness was ultimately determined according to thresholds set forth by the Commission for Macroeconomics and Health and World Health Organization in 2002, which stipulate that an intervention is “highly cost-effective” and “cost-effective” at 1 and 3 times the value of per capita gross domestic product per DALY averted, respectively. To facilitate comparison with alternative resource uses, we additionally compared findings against those available in the literature, including the Disease Control Priorities Project, third edition that highlights low-cost, high-priority interventions for key regions for the world, including sub-Saharan Africa.

## Results

### Costs

Table 1 presents program costs by phase and activity for the 10-year time horizon taking into account the rollout plan shown (Table 2). The mean annualized cost per district over the 10 years of the MOTECH-modeled program is US $237,745: US $4,110 per district for central development and program planning activities over 6 months, US $44,138 to initiate the program over 6 months, and US $17,918 to sustain the program annually. Development phase costs, which include program design, telecommunications negotiation, and system development from existing technology, are all incurred at a central level. Personnel costs accounted for 85% (US $596,955/US $698,647) of total costs in that phase. Start-up costs include some central- and some district-level activities, such as district profiling, content localization for health messaging, customer support, training for all health workers and district health teams, community mobilization through durbars or community meetings, and more. The main cost drivers in this phase are mobile phone device purchase and district-wide training, which together account for over 65% (US $4,878,377/US $7,503,508) of total start-up phase costs. During the years of program implementation, costs at the district level are focused on technical working group meetings, monitoring and evaluation, refresher and new hire training, and technical support and maintenance of the system. Costs in this phase are also driven primarily by personnel (47%, US $11,218,454/US $24,135,461), equipment (20%, US $4,928,480/ US$24,135,461) including phone replacement and annual registry replacement costs as well as airtime and data costs (10%, US $2,343,275/US$24,135,461) associated with health messaging and appointment reminders.

### Health Effects

Multimedia Appendix 2 present data on health effects adjusted for district rollout and duration of program implementation. Based on incremental changes in facility delivery, skilled birth attendance, and measles coverage, a total of 6298 maternal deaths, 33,797 child deaths, and 19,811 stillbirths would be averted over the 10-year analytic time horizon. This would correlate to a yearly average decrease in the <5 mortality rate by 4 per 1000 births. While the program is not anticipated to save lives in the first year of implementation, a total of 483 lives would be saved in Year 2; 59.6% (288/483) of these are child deaths averted, 31.5% (152/483) stillbirths averted, and 8.9% (43/483) deaths averted. By Year 10, when implementation would have occurred in all 170 districts for at least 7 years, a total of 11,938 lives would be saved. Cumulative lives saved over the 10-year analytic time horizon are anticipated to correspond to 1,550,028 DALYs averted.

### Incremental Cost-Effectiveness

Multimedia Appendix 3 presents data on the incremental cost-effectiveness of MOTECH based on deterministic values draw from 10 year estimates of costs and consequences implementation across 170 districts. The deterministic costs per DALY averted decreased over time with increasing scale and coverage from US $173.62 in Year 1 to US $6.54 in Year 10. Probabilistic estimates of the total cost per DALY averted and total cost per death averted after 10 years are US $20.92 (95% CI US $20.34-$21.55) and US $586.72 (95% CI US $569.36-$604.09), respectively.

### Uncertainty Analyses

**Figure 1:**
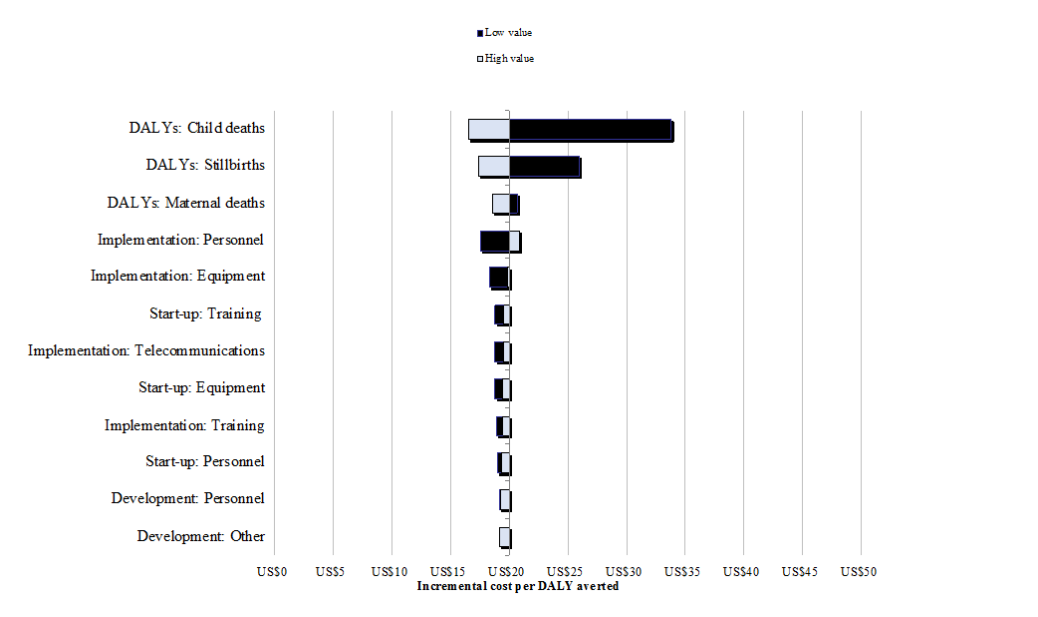
Tornado diagram showing one-way sensitivity analyses on key drivers of cost-effectiveness.DALYs: disability-adjusted life years.

**Figure 2:**
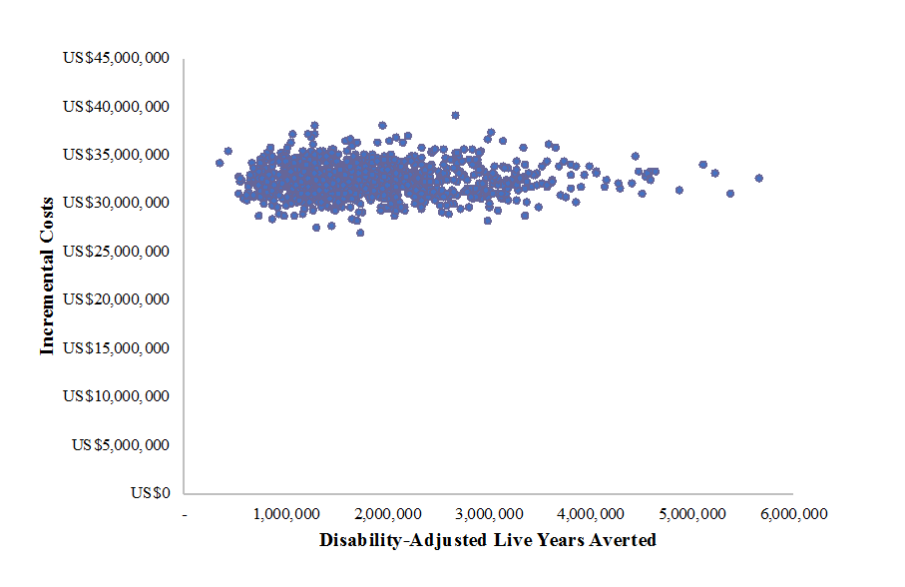
Cost-effectiveness plane of incremental program costs and disability-adjusted life years (DALYs) averted from Mobile Technology for Community Health implementation in 170 districts from 2015-2024.

**Figure 3:**
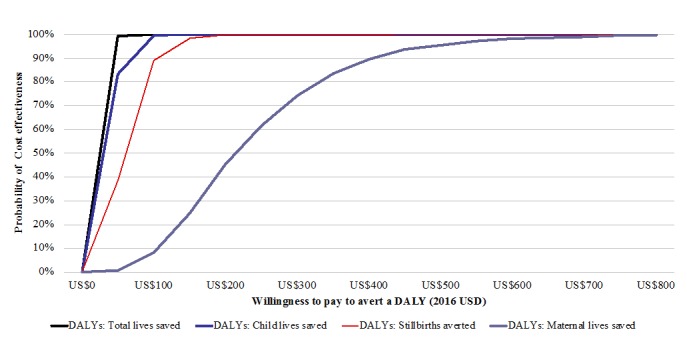
Incremental cost-effectiveness acceptability curves of Mobile Technology for Community Health implementation from 2015-2024 versus status quo in 170 districts of Ghana. DALYs: disability-adjusted life years.

Figure 1 presents findings from a 1-way sensitivity analysis. Estimates of the cost per DALY averted are most sensitive to changes in health effects parameters, including estimates of lives saved among children, stillbirths, and mothers as well as personnel costs during implementation. Probabilistic sensitivity analyses sought to explore the effects of varying multiple parameters simultaneously (Multimedia Appendix 3). The cost-effectiveness plane presented in Figure 2 depicts the incremental costs and consequences of 1000 simulations, all of which fall in the northeast quadrant. Figure 3 presents incremental CEACs of MOTECH implementation from 2015-2024 versus status quo in 170 districts of Ghana for varying denominators of DALYs based on cumulative lives saved versus DALYs based on child, maternal, or stillbirths averted. With a gross national income per capita for 2015 of US $1,480 as the threshold, program activities have a 100% probability of being cost-effective. At lower willingness-to-pay thresholds, MOTECH has a 90% probability of being cost-effective above a willingness-to-pay threshold of US $400 when maternal lives saved are used to generate DALYs and a 100% probability of being cost-effective above a willingness-to-pay threshold of US $50 when cumulative lives saved are used to generate DALYs.

## Discussion

### Principal Findings

This study assessed the costs and consequences of implementing an mHealth program–dually providing an interactive voice message system to pregnant and new mothers as well as a health record and reminder system for use by frontline health workers–as compared to the pre-existing health services over 10 years in nearly 80% of districts across Ghana. If implemented across Ghana through 170 districts over 10 years, MOTECH could potentially save an estimated 59,906 lives at a total 10-year cost of US $32 million. Estimates of the total annualized cost per district of US $66,166 were driven by start-up costs including provider training and equipment. The cost to sustain the program once implemented would be US $17,618 on average annually per district. In Ghana’s 2013 government budget, US $264.5 million was earmarked for the health sector of which 72% was specifically directed for primary care [29]. Assuming this directive funding is continued in the modeled MOTECH timeframe with equal allocation to 170 districts, then developing and starting the MOTECH program would equate to 4% of the district budget, and sustaining the program would be less than 1.6%.

Efforts to determine the value for money of MOTECH suggest that program activities are highly cost-effective at a total cost per DALY averted of US $20.94 (95% CI US $20.34-$21.55) over 10 years–an estimate that falls well below the gross national income for Ghana of US $1,480 and compares favorably with alternative resource uses [30,31]. As implementation at scale continues and integration with existing or planned digital health solutions occurs it is probable that further declines in costs could be attained, corresponding to even greater improvements in cost-effectiveness. Alternatively, it is also possible that 1 of MOTECH’s components (CDA or Mobile Midwife) could be scaled in isolation, which may also affect value for money estimates. Our analyses did not differentiate the costs and consequences of the subcomponents of MOTECH. However, messaging has been shown to be an effective strategy for client engagement across a number of geographies [7,32,33], and evidence is emerging on its probable cost-effectiveness. In South Africa, a small-scale deployment of MAMA maternal messaging forecasted to scale across 1 province over 5 years reported a US $200 cost per DALY averted [34]—a finding nearly 10 times higher than that estimated for Ghana when maternal messaging is provided in conjunction with the supply-side CDA.

Estimates of the value for money of initiatives similar to CDA that digitize patient health records and send alerts and reminders are not yet available in the literature. While such programs may have some effects on improved efficiencies in reporting, in practice, these can be difficult to translate into measurable health outcomes. Mobile e-Tracker, a new feature of the national population health monitoring DHIS2 system in Ghana, is an example of 1 initiative underway that may ultimately take the place of CDA at scale should GHS not opt to expand and sustain MOTECH as this analysis considers. E-Tracker aims to digitize individual client data for aggregation at district, regional, and national levels, a step that may ease reporting and improve the national health information system. To date, implementation has occurred in 200 hospitals around the country, which process, on average, 1000 new admissions per day using ICD-10 codes [25]. Whether e-Tracker will be expanded to the community level and taken up by frontline health workers remains to be seen. In the event that it is, our analyses highlight the potential synergistic effect that concurrent implementation with a demand-generating initiative like Mobile Midwife may have. Early findings on the cost-effectiveness from other programs, such as the Smart Registries Project (OpenSRP), led by the WHO, or the mCARE program in Bangladesh, which have drawn inspiration from the MOTECH strategy of combined client and health worker engagement, echo these results, suggesting value for money particularly over time and with sufficient scale [35].

### Comparison With Alternative Resource Uses

Data on the cost-effectiveness of interventions to improve MNCH coverage are emerging [20]. Incremental cost-effectiveness ratios from these studies range from US $79 per DALY averted, for a 10-year forecasted program that supports delivery care in Zambia and US $214 per DALY averted, for a home-care service package that includes community mobilization and health system strengthening aspects in Bangladesh, to US $302 per DALY averted, for a women’s group quality improvement collaborative to improve birth outcomes in rural Nepal [20,36-38]. When compared against studies that report findings on the cost per life saved, MOTECH similarly falls well below program alternatives with a cost per death averted of US $586.72 (95% CI US $569.36-$604.09). The quality improvement collaborative in Nepal reported US $8,670 per life saved, the Zambian program supporting delivery care reported US $1,988 per life saved, and a maternal health mobile unit providing outreach services in The Gambia reported a cost per life saved range of US $1,380-$6,414 [20,37-39]. Collectively, these studies suggest that MOTECH, even in its earliest years of implementation, where the cost per DALY averted is US $173.62, is likely to provide good value for money.

In the context of other digital health programs, only a dozen peer-reviewed articles comprise the body of evidence on the value for money of mHealth solutions, including cost-effectiveness analyses (5 studies [40-44]), cost-utility analyses (2 studies [45,46]), and cost-benefit analyses (4 studies [47-50]). Ours is the first effort to forecast costs and consequences of scaling up an mHealth solution and the only study to date of an integrated behavior change communication and service delivery mHealth solution. The effectiveness results may be further improved if the program implementation targeted districts or regions with a worse health status first, in order to have longer implementation and achieve greater effects in those areas.

### Limitations

This study encompasses several important limitations. Efforts to determine the economic costs of MOTECH implementation were conducted from a program perspective, drawing upon data obtained from a single district. While these costs have been modified to best model potential program expenditure, they do not represent planned government resource use or cost structures. In particular, personnel costs are based on Grameen Foundation personnel in Accra and in Gomoa West rather than GHS personnel or salary costs, as this level of granularity in government expenditure was not available at the time of the analysis. To account for these limitations, we achieved a granular level of cost and activity data that are related to the number of nurses, CHPS facilities, and health centers, which we used to vary mHealth program costs across different districts to best estimate national-scale costs in Ghana. Further sensitivity analyses were used to characterize the effects of changes to these parameters on emerging cost-effectiveness estimates. To forecast the costs of scaling up program activities to 170 districts, we drew from the most recent census data available, the 2010 Ghana Population and Housing Report, and generated a rollout plan to estimate associated costs for these 170 districts [23]. In 2012, 46 new districts were created; however, in the absence of updated population or health facility data, the model could not adequately estimate the program scaling costs for these presently defined districts [24]. Finally, it is noteworthy that our analyses did not include costs incurred by pregnant and postpartum women to seek care or to the health system to collect data using CDA or accommodated forecasted increases in utilization stemming from increased demand. Future evaluations of digital health programs should plan economic evaluations from the outset that can prospectively track costs from a societal perspective.

To model health effects, we drew from data collected as part of a randomized controlled trial exploring the effects of MOTECH versus status quo activities on key MNCH outcomes, including skilled birth attendance, facility delivery, and immunizations, conducted in Gomoa West led by the Healthcare Innovation Technology Lab in partnership with the University of Ghana School of Public Health. In brief, methodological limitations were seen in the study design, methods for data collection (sampling, survey tool), and the analytic approach. Among these factors, limitations in the survey tool and wording of indicators as well as the decision to recruit the sample population from community outreach activities organized by GHS community health nurses versus sampling at a household population level has rendered efforts to estimate the effects of MOTECH activities on population-level changes in service utilization challenging. In an effort to work with the data available to us, we generated propensity scores based on values of education, employment, marital status, age, and income at endline. We then estimated average treatment (MOTECH intervention) effects based on nearest neighbor matching based on propensity score values. The end changes in coverage were then inputted into the LiST and used to generate lives saved and, ultimately, a years of life lost only-based DALY. For the health effects included, we further attenuated coverage gains anticipated over time across the time horizon in order to account for declines in the magnitude of gains anticipated as coverage reached thresholds of 75% and above for key interventions. To account for uncertainty in our primary effects data as well as forecasted changes over time, we conducted 1-way and probabilistic sensitivity analyses. We further presented CEACs that included cumulative lives saved as well as those based on maternal, child, and stillbirths averted.

### Conclusion

Study findings suggest that supply- and demand-side digital health initiatives like MOTECH may offer value for money when implemented over time and at scale. Further research is needed to validate underpinning assumptions and generate more robust evidence on the costs and consequence of expanding and sustaining the implementation MOTECH in Ghana.

## Appendix

Multimedia Appendix 1 Subsample of mobile midwife postpartum messages occurring in the first 1 month.

Multimedia Appendix 2 Health effects adjusted for district rollout and duration of MOTECH program implementation.

Multimedia Appendix 3 Parameters for probabilistic sensitivity analyses.

## Abbreviations

ANC: antenatal care
BMGF: Bill and Melinda Gates Foundation
CDA: Client Data Application
CEAC: cost-effectiveness acceptability curve
CHPS: Community-Based Health Planning and Services
DALY: disability-adjusted life year
DHIS: District Health Information System
GHS: Ghana Health Service
LiST: Lives Saved Tool
mHealth: mobile health
MNCH: maternal, newborn, and child health
MOTECH: Mobile Technology for Community Health
