# Supplementary material for: Mobile Technology for Community Health in Ghana: Is Maternal Messaging and Provider Use of Technology Cost-Effective in Improving Maternal and Child Health Outcomes at Scale?
Source: J Med Internet Res. 2019 Feb 13;21(2):e11268. doi: 10.2196/11268 (PMC6391645; doi:10.2196/11268)
Supplement: Multimedia Appendix 1 [file jmir_v21i2e11268_app1.pdf]

Supplementary Table 1. Sub-sample of mobile midwife postpartum messages occurring in the first 1 month

**MOTECH (GHS/Grameen Foundation): Postpartum/First Year of Life Messages**		
**Week**	**Health Topic**	**Postpartum/First Year of Life Messages**
1st wk	1st Postnatal care	The first PNC visits are very important. If for any reason you gave birth outside of a medical facility, you need to attend PNC within 24 hours of giving birth. Even if you feel fine and your baby seems well it is important to go. Because of the amount of blood lost during delivery you can become anemic or have very low blood. You will be given a dose of Vitamin A, and another one when you go to the PNC visit 2 days after the birth which will help raise your blood levels. Your baby will be weighed, measured and get their first immunizations which are very important to protect your baby against Polio and TB. You can also register your baby for a birth certificate at this time. It is best to go to PNC two times during the first week of your baby's life and again when your baby is 6 weeks old. If you haven't been yet be sure to go this week.
2nd wk	Fontenelle/ Cord Care	By now your baby's umbilical cord stump should be dry and may have fallen already. If you see any puss or redness at the area take your baby to clinic right away as infections can be dangerous to your new baby. Also, every baby has a soft spot on the top of his or her head where the bones have not yet sealed giving your baby's brain room to grow. It will start to close by itself at about 6 months. This soft spot is not an ulcer and you should not put hot compresses or any herbal medicines on it. The spot should be flat or raised a bit when you feel it. If the spot has sunk down and it feels like a hole you must go to the clinic as your baby is not well. Do not try to shape your baby's head as this is dangerous and can cause serious damage to your little one's brain.
3rd wk	Exclusive breastfeeding	Breast-fed babies are healthier, stronger and suffer fewer sicknesses than babies that are fed other foods or drinks. Breast-milk alone 8 - 12 times a day, is all baby needs to eat for the first 6 months of life. Breast milk contains water so your baby doesn't need to be given water. The right positioning during feeding is important for your baby to eat well. Your baby's whole body should be turned to face you, and you and your baby should be belly to belly. Signs that your baby is feeding well are: baby's chin is touching your breast, more of the dark skin around your nipple can be seen above your baby's mouth than below it, your baby takes long, deep sucks, and you don't feel any pain in the nipple. Both of your breasts should feel empty when your baby is finished. If you are worried that your baby is not getting enough to eat, don't be afraid to ask your midwife. They can help you so that you are sure that your little one gets all they need to grow healthy and strong.

4th wk	Malaria	Malaria is very dangerous to a newborn baby and is caused by the bite of mosquitos. Be sure that you and your baby sleep under an insecticide treated mosquito net. Be sure that evening time and early dawn your baby is protected from mosquitos by dressing them in something light that covers their legs, feet and arms. Remember to keep your room mosquito free with sprays or by burning coils. Also clearing bushes around the house, and covering water containers will help control mosquitos. If baby has a fever, is not interested in eating, not sucking well, or spitting up with force, or their eyes or skin look yellow, do not wait to take them to the clinic. Early treatment can save your baby's life.
5th wk	Infection Control/ hygiene	Good hand washing practices can prevent the spread of diseases such as polio, rotovirus, typhoid and cholera and eye infections. It is always important to keep your home environment clean, but the best way to keep germs from causing a problem for your baby is by washing your hands. Wash your hands with soap and clean water, rubbing them well to remove the germs. Always wash your hands before handling your newborn, and after changing your baby's nappies. And of course, wash hands after toileting, after handling raw food and always before cooking or eating. Don't be afraid to ask others to wash their hands before holding or touching your new baby as well. We can't protect babies from everything that might make them sick but hand washing is an important step to help keep your baby and the rest of your family healthy.
6th wk	Immunization	Immunization gives the body protection from certain diseases. An immunization tells the body to create antibodies which are like soldiers that fight off the disease. This week your baby needs to receive immunization against Tetanus/diphtheria/whooping cough (DPT), influenzaB(HiB), Hepatitis B, Polio, Rotavirus and Pneumonia. You will also have your blood checked to see if you have low blood, and have time to talk about problems you may be having with breastfeeding or anything else. Immunizations are free under NHIS, so go to a NHIS accredited facility. Immunizations are not harmful and can protect your baby from diseases that can kill them.
7th wk	MEN: Contraception/Postnatal clinic	MEN: Your wife needs to report to PNC around now to make sure she is fine, after giving birth. One thing that should be talked about at PNC is contraception in order to space your children at least 2 years apart. Contraception methods are a personal choice for every couple and it gives your wife’s body enough time to fully recover before getting pregnant again. Using contraception is not so that your wife can be promiscuous or go outside of your marriage. Getting pregnant again too soon can put her life at risk, as her blood levels may still be low. She can also bleed too much with the next delivery. While breastfeeding she may be protected from getting pregnant for some months, but she can still get pregnant even if breastfeeding. Speak to your doctor about a contraception method that will work for you and your wife, take time to decide and start the method as soon as your wife is no longer exclusively breast feeding.

8th wk	Eye infections	Eye infections are common in children and are very contagious. When your baby wakes up from a sleep you may notice their eyes are either crusty or sticky. To help your baby open the eyes when they are sticky, use clean warm water on a piece of cotton wool to gently wipe the eye. Take your baby to the clinic if the whites of the eyes are pink, red or yellowish. Infections left untreated can cause blindness or seriously damage your baby's eyes so don't try to solve the problem yourself. Seek medical advice without delay.
