# Supplementary material for: Mobile Technology for Community Health in Ghana: Is Maternal Messaging and Provider Use of Technology Cost-Effective in Improving Maternal and Child Health Outcomes at Scale?
Source: J Med Internet Res. 2019 Feb 13;21(2):e11268. doi: 10.2196/11268 (PMC6391645; doi:10.2196/11268)
Supplement: Multimedia Appendix 2 [file jmir_v21i2e11268_app2.pdf]

Multimedia Appendix 2. Health Effects Adjusted for District Rollout and Duration of MOTECH Program Implementation.

Inter-vention	Changes in coverage by intervention type			2015	2016	2017	2018	2019	2020	2021	2022	2023	2024
	Annual increases in coverage until 75% of population reached*	Annual increase in coverage between 75 – 90%	Annual increase in coverage between 90 – 99%	Modeled coverage increase by base case program coverage effect for districts, adjusted for time and level of coverage in the population									
**Facility delivery**	**Base: 10.4%**<br>Upper: 18.8%<br>Lower: 2.0%	**5.20%**<br>9.42%<br>0.99%	**2.60%**<br>4.71%<br>0.49%	**57.1%**	**67.5%**	**77.9%**	**83.1%**	**88.3%**	**90.9%**	**93.5%**	**96.1%**	**99.0%**	**99.0%**
**SBA**	**Base: 11.02%**<br>Upper: 19.4%<br>Lower: 2.6%	**5.51%**<br>9.72%<br>1.30%	**2.75%**<br>4.86%<br>0.65%	**58.7%**	**69.7%**	**80.7%**	**86.2%**	**91.8%**	**94.5%**	**97.3%**	**99.0%**	**99.0%**	**99.0%**
**Measles**	**Base: 6.0%**<br>Upper: 10.3%<br>Lower: 1.7%	**2.99%**<br>5.14%<br>0.84%	**1.50%**<br>2.57%<br>0.42%	**91.0%**	**92.5%**	**94.0%**	**95.5%**	**97.0%**	**98.5%**	**99.0%**	**99.0%**	**99.0%**	**99.0%**
**Number of Lives Saved**													
Maternal				0	43	149	288	395	636	950	1,224	1,283	1,330
Child				0	288	1,001	1,941	2,668	3,749	5,038	6,119	6,397	6,596
Stillbirth				0	152	534	1,043	1,444	2,119	2,957	3,678	3,872	4,012
**Total Lives Saved**				**0**	**483**	**1,684**	**3,272**	**4,507**	**6,504**	**8,945**	**11,021**	**11,552**	**11,938**

*Data drawn from the independent evaluation of MOTECH in Gomoa West led by the Healthcare Innovation Technology Lab in partnership with University of Ghana School of Public Health
