# Supplementary material for: Mobile Technology for Community Health in Ghana: Is Maternal Messaging and Provider Use of Technology Cost-Effective in Improving Maternal and Child Health Outcomes at Scale?
Source: J Med Internet Res. 2019 Feb 13;21(2):e11268. doi: 10.2196/11268 (PMC6391645; doi:10.2196/11268)
Supplement: Multimedia Appendix 3 [file jmir_v21i2e11268_app3.pdf]

Multimedia Appendix 3. Parameters for probabilistic sensitivity analyses.

Deterministic values draw from 10 year estimates of costs and consequences of MOTECH implementation across 170 districts.

Parameter	Deterministic Base case	High	Low	Distribution	Probabilistic	Distribution parameters a	b	Mean c	SD
**Development**									
Program Design	$36,800	$46,000	$34,872	Gamma	$36,396	174.977	210	36,800.00	2,782.00
Telecommunications	$23,480	$24,170	$23,232	Gamma	$23,554	10,020.685	2	23,480.12	234.56
Technology	$16,907	$18,599	$16,011	Gamma	$16,793	682.730	25	16,906.67	647.04
Vehicle Maintenance	$8,910	$9,515	$8,443	Gamma	$8,800	1,105.579	8	8,910.07	267.97
Office Maintenance	$15,595	$17,156	$14,769	Gamma	$15,945	682.730	23	15,595.37	596.86
Personnel and Benefits	$596,955	$746,193	$553,634	Gamma	$652,325	153.771	3,882	596,954.68	48,139.83
Subtotal Development	***$698,647***	***$861,633***	***$650,960***	***Gamma***	***$659,540***	***175.961***	***3,970***	***698,646.91***	***52,668.26***
**Start Up**									
District Profiling	$150,919	$198,874	$102,963	Gamma	$135,445	39.616	3,810	150,918.69	23,977.70
Content Localization	$30,690	$38,363	$23,018	Gamma	$30,798	64	480	30,690	3,836
Equipment	$2,471,799	$2,926,175	$1,799,322	Gamma	$2,492,618	77	32,107	2,471,799	281,713
Customer Support	$249,746	$312,182	$187,309	Gamma	$263,174	64	3,902	249,746	31,218
Training	$2,406,578	$3,008,222	$1,804,933	Gamma	$2,254,763	64	37,603	2,406,578	300,822
Community Mobilization / Marketing	$627,638	$784,547	$470,728	Gamma	$551,917	64	9,807	627,638	78,455
Partnership Building	$10,449	$13,061	$7,837	Gamma	$12,241	64	163	10,449	1,306
Vehicle Maintenance	$172,082	$215,102	$129,061		$187,357	64	2,689	172,082	21,510
Office Maintenance	$86,277	$107,846	$64,707	Gamma	$87,258	64	1,348	86,277	10,785
Telecommunications	$77,166	$95,933	$57,874	Gamma	$73,622	66	1,173	77,166	9,515
Technology	$26,318	$32,898	$19,739	Gamma	$25,817	64	411	26,318	3,290
Personnel and Benefits	$1,193,847	$1,497,592	$895,385	Gamma	$1,121,524	63	18,986	1,193,847	150,552
Subtotal Start up	***$7,503,508***	***$9,230,796***	***$5,562,878***	***Gamma***	***$8,574,782***	***67***	***112,061***	***7,503,508***	***916,980***
**Implementation**									
Technical Groups	$17,810	$22,263	$13,358	Gamma	$17,684	64	278	17,810	2,226
M&E	$1,700,567	$2,125,709	$1,275,425	Gamma	$1,832,128	64	26,571	1,700,567	212,571
Continued Training	$2,140,605	$2,675,757	$1,605,454	Gamma	$1,885,825	64	33,447	2,140,605	267,576
Equipment & Materials	$4,928,480	$6,054,802	$3,526,178	Gamma	$5,170,477	61	81,084	4,928,480	632,156
Vehicle Maintenance	$1,078,131	$1,347,663	$808,598	Gamma	$972,268	64	16,846	1,078,131	134,766
Field Office Maintenance	$209,521	$211,407	$164,481	Gamma	$200,623	319	657	209,521	11,732
Office Maintenance	$257,036	$317,618	$192,777	Gamma	$275,448	68	3,790	257,036	31,210
Telecommunications	$2,343,275	$2,929,093	$1,757,456	Gamma	$2,544,513	64	36,614	2,343,275	292,909
Technology Support and Maintenance	$241,582	$301,977	$181,186	Gamma	$245,022	64	3,775	241,582	30,198
Personnel and Benefits	$11,218,454	$14,023,06	$8,413,841	Gamma	$11,327,320	64	175,288	11,218,454	1,402,307

		8							
Subtotal Implementation	***$24,135,461***	***$30,009,358***	***$17,938,754***	***Gamma***	***$20,659,061***	***64***	***377,296***	***24,135,461***	***3,017,651***
**Total Societal Cost**	**$32,337,616**	**$40,101,787**	**$24,152,592**	**Gamma**	**$32,919,189**	**66**	**491,643**	**32,337,616**	**3,987,299**
**LIVES SAVED**						Media ln(x)	SD ln(x)		
Maternal	6,298	8,519	1,133	Lognormal	4,625	9	0.51	6,298	
Children < 5 years	33,797	43,390	8,356	Lognormal	15,686	10	0.42	33,797	
Stillbirths	19,811	26,079	4,407	Lognormal	15,025	10	0.45	19,811	
Total Lives Saved	***59,907***	***77,988***	***13,896***	***Lognormal***	***66,824***	***11***	***0.44***	***59,907***	
**DALYs**									
Maternal	149,409	202,100	26,875	Lognormal	109,810	12	0.51	149,409	
Children < 5 years	968,589	1,243,528	239,474	Lognormal	1,058,249	14	0.42	968,589	
Stillbirths	567,767	747,402	126,298	Lognormal	627,775	13	0.45	567,767	
Total DALYS Averted	***1,685,765***	***2,193,029***	***392,647***	***Lognormal***	***2,081,914***	***14***	***0.44***	***1,685,765***	
